# Effect of one-step recrystallization on the grain boundary evolution of CoCrFeMnNi high entropy alloy and its subsystems

**DOI:** 10.1038/srep22306

**Published:** 2016-02-29

**Authors:** Bo-Ru Chen, An-Chou Yeh, Jien-Wei Yeh

**Affiliations:** 1 Department of Materials Science and Engineering, National Tsing Huang University, Hsinchu, Taiwan 30013, ROC

## Abstract

In this study, the grain boundary evolution of equiatomic CoCrFeMnNi, CoCrFeNi, and FeCoNi alloys after one-step recrystallization were investigated. The special boundary fraction and twin density of these alloys were evaluated by electron backscatter diffraction analysis. Among the three alloys tested, FeCoNi exhibited the highest special boundary fraction and twin density after one-step recrystallization. The special boundary increment after one-step recrystallization was mainly affected by grain boundary velocity, while twin density was mainly affected by average grain boundary energy and twin boundary energy.

The concept of grain boundary engineering (GBE) has been previously proposed by Watanabe^1^, and its aim is to improve resistance to oxidation, corrosion, creep, and the propagation of cracks in materials^2^,^3^,^4^,^5^,^6^ by controlling the grain boundary character distribution (GBCD) with high special boundary population and broken interconnectivity of high angle grain boundaries (HAGBs). Special boundaries can be expressed by coincidence site lattice (CSL) boundaries with low-Σ values (3 ≤ Σ ≤ 29), where Σ is the reciprocal density of coinciding sites. Compared to HAGBs, special boundaries have a relatively small excess volume because of their geometrical arrangements, and thus possess lower boundary energy. The lower grain boundary energy is the underlying mechanism responsible for the improved properties because of its corresponding low diffusivity^7^,^8^.

GBE has been applied on various alloy systems, including FCC austenitic stainless steels^4^,^9^,^10^ and nickel-based superalloys^2^,^3^,^5^,^11^ that possess relatively low stacking fault energy. One-step recrystallization is one of the four categories of GBE processes for alloys with relatively low stacking fault energy, as summarized by Randle^12^. For FCC alloys, special boundaries are mainly composed of Σ3^n^s boundaries, especially for twin Σ3 boundaries^12^,^13^. Based on the growth accident model for annealing twin formation^14^, Pande *et al.*^15^,^16^ have stated that twin density depends on the average grain size, average grain boundary energy, and twin boundary energy of the material. Cahoon *et al.*^17^ have further stated that applied strain can affect twin density. For special boundary reactions, Randle has proposed that when strain induced boundary migration takes place, non-twin Σ3 boundaries with high mobility can increase special boundary fraction by the Σ3 regeneration mechanism, which indicates that the interactions between Σ3^n^s can produce Σ3s and further be incorporated into a grain boundary network, e.g. 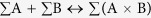 or 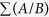
12. In addition, Hwang *et al.*^18^ have observed another two types of special boundary interactions by *in-situ* electron backscatter diffraction (EBSD): (1) HAGB dissociation, which indicates the rapid migration of specific HAGBs being dissociated into Σ3 boundaries because of a growth accident, and (2) annihilation of the special boundary by HAGB migration. Each of these theories have helped to establish the underlying mechanisms for GBCD during GBE processes.

The high entropy alloy (HEA) system proposed by Yeh *et al.*^19^ has drawn much attention recently. HEAs can exhibit sluggish diffusion and severe lattice distortion effects^20^. Sluggish diffusion effects can hinder the grain boundary migration^19^, and severe lattice distortion effects can impede dislocation movement and decrease stacking fault energy^21^,^22^. Both of these HEA effects can have significant effects on the GBCD. An equiatomic CoCrFeMnNi high entropy alloy has been reported to possess a single-phase FCC crystal structure^23^. Furthermore, within subsystems of CoCrFeMnNi, two binary alloys (FeNi and CoNi), five ternary alloys (FeCoNi, CrFeNi, FeMnNi, CoCrNi, and CoMnNi), and three quaternary alloys (CoCrFeNi, CoFeMnNi, and CoCrMnNi) have also been reported to possess single-phase FCC crystal structures^24^. These single-phase FCC alloys are ideal subjects for investigating the differences in evolutions of GBCD between higher-order and lower-order alloy systems during the GBE process. Furthermore, HEAs are reported to possess lower stacking fault energy than those of conventional alloys, because the increase in severe lattice distortion can increase the matrix energy and decrease the potential difference between stacking fault and matrix^21^,^22^,^24^. For example, Wu *et al.*^24^ have reported that the stacking fault energy of CoCrFeNi is lower than that of FeCoNi. Additions of Mn can decrease the stacking fault energy of Ni^25^. The stacking fault energies of CoCrFeNi and CoCrFeMnNi have been measured by Zaddach *et al.*^22^ using the x-ray diffraction method coupled with first principle calculations, and it has been reported that CoCrFeMnNi possesses lower stacking fault energy than that of CoCrFeNi. This suggests that HEAs may form annealing twin ∑3 boundaries more easily during GBE processes.

In this research, one-step recrystallization was conducted on equiatomic FeCoNi, CoCrFeNi, and CoCrFeMnNi alloys. Analysis was focused on the evolutions of GBCD in order to investigate its correlation with increasing alloying complexity from 3 to 5 elements. The present work is the first to study the effects of grain boundary engineering processes on the grain boundary evolution of high entropy alloy comparing with those of lower-order alloy systems. The underlying mechanisms of GBCD of high entropy, medium entropy, and lower entropy alloys have been discussed in this article.

## Results and Discussions

Samples of CoCrFeMnNi, CoCrFeNi, and FeCoNi were arc melted and homogenized first by heat treatment before a 50% reduction in thickness. All three alloys were then subjected to annealing at different conditions in order to compare their grain boundary velocity; details are described in the methods section. The melting temperatures of CoCrFeMnNi, CoCrFeNi, and FeCoNi were previously reported to be 1289 °C, 1420 °C, and 1440 °C, respectively^26^. The recrystallization temperatures determined in this work for CoCrFeMnNi, CoCrFeNi, and FeCoNi were 900 °C, 800 °C, and 700 °C, respectively. The annealing temperature for each alloy is at and above the recrystallization temperature for each alloy.

**Figure 1:**
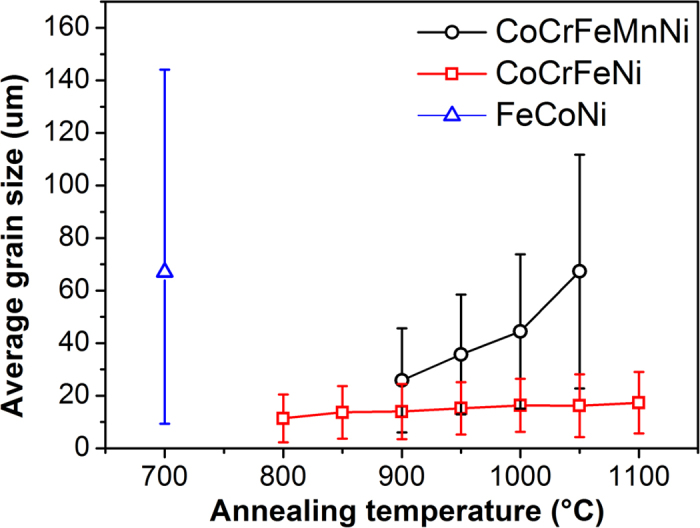
Grain size distribution of CoCrFeMnNi/CoCrFeNi/FeCoNi after REC (1 h annealing).

Figure 1 shows the grain size analysis after 1h annealing; FeCoNi possesses the fastest grain boundary velocity, and CoCrFeNi possesses the slowest grain boundary velocity among the tested alloys. In addition, the grain growth of CoCrFeNi seems to be suppressed at higher annealing temperatures. CoCrFeMnNi possesses a medium grain boundary velocity. These phenomena may be directly related to the alloying element; it indicates that the increase in Cr content can decrease grain boundary velocity. For CoCrFeMnNi and CoCrFeNi, the standard deviation of grain size was much smaller than the average grain size, so grains were homogeneous. The average grain aspect ratios of CoCrFeMnNi and CoCrFeNi were between 1.62 and 1.73; this indicates that the grains were nearly equiaxed. For FeCoNi, the standard deviation of grain size was very close to the average grain size, indicating that grains were less homogenous. The average grain aspect ratio of FeCoNi was between 1.76 and 1.87, which suggests that the grains were slightly less equiaxed.

**Figure 2:**
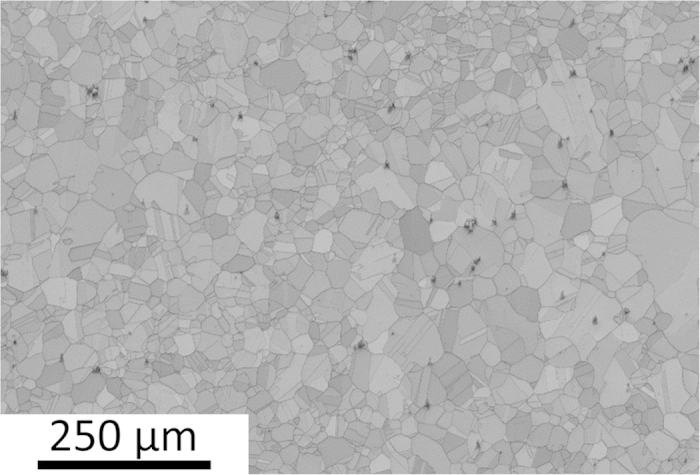
Mn-Cr oxides in CoCrFeMnNi alloy and its composition (dark phase).

**Figure 3:**
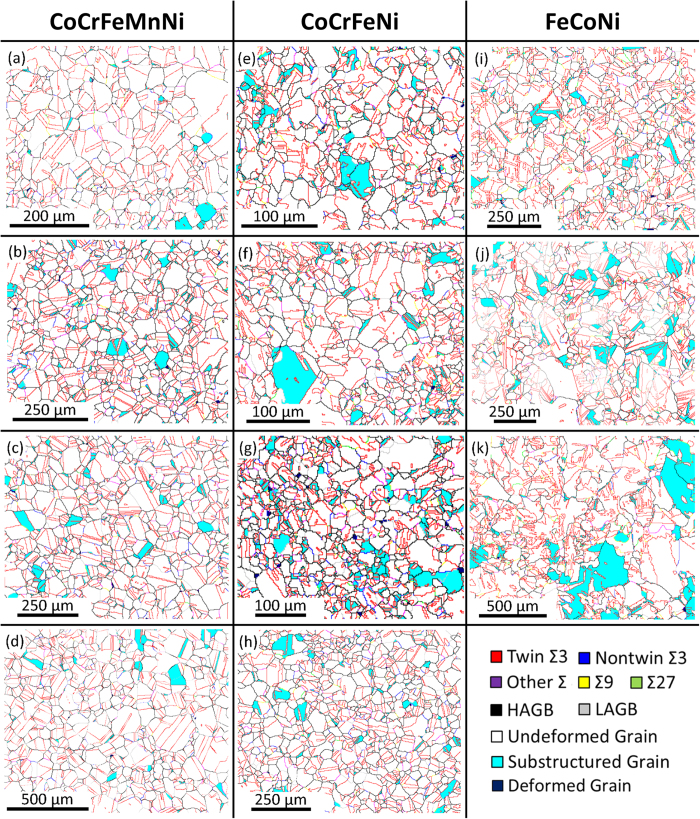
Remained strain analysis of REC for CoCrFeMnNi: (**a**) 900 °C/1 h, (**b**) 950 °C/1 h, (**c**) 1000 °C/1 h, (**d**) 1050 °C/1 h; CoCrFeNi: (**e**) 800 °C/2 h, (**f**) 800 °C/6 h, (**g**) 1100 °C/1.5 h, (**h**) 1100 °C/2 h; FeCoNi: (**i**) 800 °C/20 min, (**j**) 800 °C/30 min, (**k**)700 °C/1 h.

**Figure 4:**
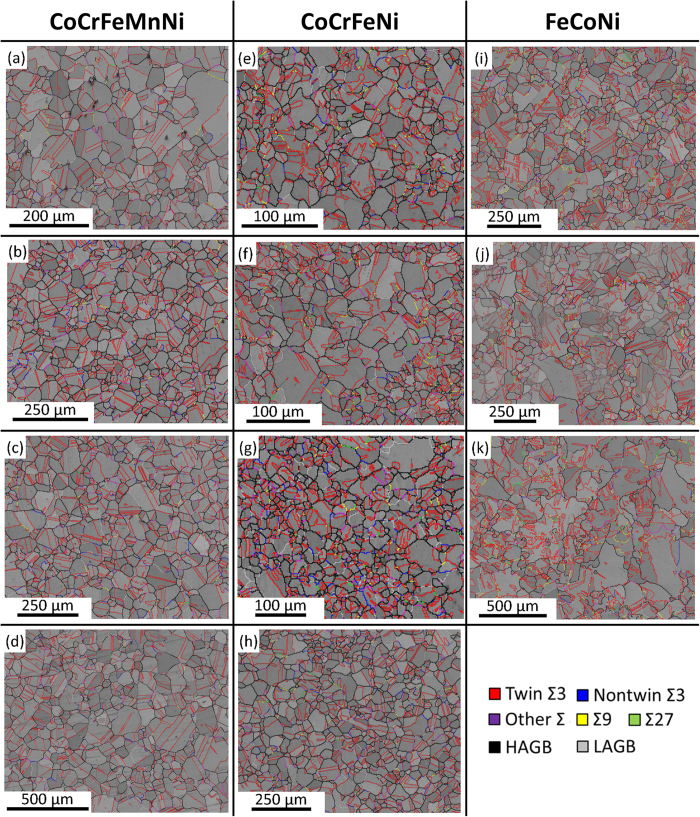
GBCD of REC for CoCrFeMnNi: (**a**) 900 °C/1 h, (**b**) 950 °C/1 h, (**c**) 1000 °C/1 h, (**d**) 1050 °C/1 h; CoCrFeNi: (**e**) 800 °C/2 h, (**f**) 800 °C/6 h, (**g**) 1100 °C/1.5 h, (**h**) 1100 °C/2 h; FeCoNi: (**i**) 800 °C/20 min, (**j**) 800 °C/30 min, (**k**)700 °C/1 h.

It should be noted that minor and well-dispersed Mn-Cr oxides with an average size of 8.94 μm were present in CoCrFeMnNi, as shown in Fig. 2. The composition of the Mn-Cr oxides is listed in Table 1. The presence Mn-Cr oxides in CoCrFeMnNi were previously reported by Tasan *et al.*^27^. It appears that it is difficult to eliminate these Mn-Cr oxides in CoCrFeMnNi, and they might have hindered the migration of grain boundaries. Nevertheless, CoCrFeMnNi showed a homogeneous grain size distribution after one-step recrystallization (REC). The ratios of recrystallization temperature over melting temperature for CoCrFeMnNi, CoCrFeNi, and FeCoNi are 0.75, 0.63, and 0.57, respectively. These ratios increased with increasing alloying elements, which can be caused by severe lattice distortion and sluggish diffusion, which impede dislocation movement. Table 2 shows the REC results of CoCrFeMnNi, CoCrFeNi, and FeCoNi with varying grain size. Figures 3 and 4 show the remained strain analysis by misorientation distribution and the GBCD of the tested alloys, respectively.

According to the remained strain analysis of CoCrFeMnNi as shown in Fig. 3(a–d) and Table 2, most of the grains are free of strain (undeformed) after REC, and only a small fraction of substructured grains were observed. Figure 4(a–d) show the GBCD of CoCrFeMnNi; some boundaries were pinned by Mn-Cr oxides, which may have slowed down the grain boundary velocity and hindered the migration of HAGBs and special boundaries, and thus decreased the frequency of boundary interaction. Only a small fraction of Σ3s are formed by Σ3 regeneration, which indicates that Σ3s are mainly formed by HAGB dissociation. According to Table 2, most special boundaries are composed of twin Σ3s, while non-twin Σ3s, Σ9s, and Σ27s occupy only a small boundary fraction and decrease slightly with increasing grain size. The special boundary fraction also shows no significant change during grain growth, which indicates that HAGB dissociation counterbalances with Σ3 annihilation for CoCrFeMnNi during REC.

Figure 3(e–h) show the remained strain analysis of CoCrFeNi after REC. Its condition was similar to that of CoCrFeMnNi, as most of the grains were undeformed and only a small fraction of substructured grains were observed. According to the GBCD of CoCrFeNi, shown in Fig. 4(e,f) and Table 2, only a small fraction of Σ3s were formed by Σ3 regeneration, which indicates that Σ3s are mainly formed by HAGB dissociation. However, the special boundary fraction showed a decreasing trend during grain growth, which indicates that Σ3 annihilation occurs for CoCrFeNi.

According to the remained strain analysis of FeCoNi, shown in Fig. 3(i–k), most of the grains were also free of strain, similar to those observed in CoCrFeMnNi and CoCrFeNi. The GBCD of FeCoNi after REC shows that the frequency of Σ3 regeneration is higher than that of CoCrFeMnNi and CoCrFeNi, as outlined in Table 2 and Fig. 4(i–k). The Σ9 boundary fraction showed an increasing trend during grain growth, much higher than that of CoCrFeMnNi and CoCrFeNi, due to the increased frequency of Σ3 regeneration. The special boundary fraction showed a significant increase during grain growth, which indicates that both HAGB dissociation and Σ3 regeneration are the main boundary interaction mechanism.

Comparing the results of GBCD for the three alloys after REC, as seen in Table 2, FeCoNi exhibited the highest special boundary fraction, while CoCrFeMnNi exhibited the lowest special boundary fraction. FeCoNi showed a positive special boundary increment with grain growth, while CoCrFeNi showed a negative trend and CoCrFeMnNi exhibited a constant value. Furthermore, both special boundary increment and boundary interaction appear to show positive correlations to grain boundary velocity. It is possible that the higher grain boundary velocity of FeCoNi during one-step recrystallization increases the frequency of boundary interaction, especially for growth accident. As a result, there is a large increase in frequency of HAGB dissociation, while a small increase in the frequency of Σ3 regeneration also occurs. As the fractions of twin Σ3 boundaries are increased, the frequency of Σ3 regeneration can be further increased, since special boundaries become easier to meet up. Because FeCoNi possesses the highest grain boundary velocity, HAGB dissociation takes an important role and introduces more Σ3s into the grain boundary network, which increases the frequency of Σ3 regeneration.

**Figure 5:**
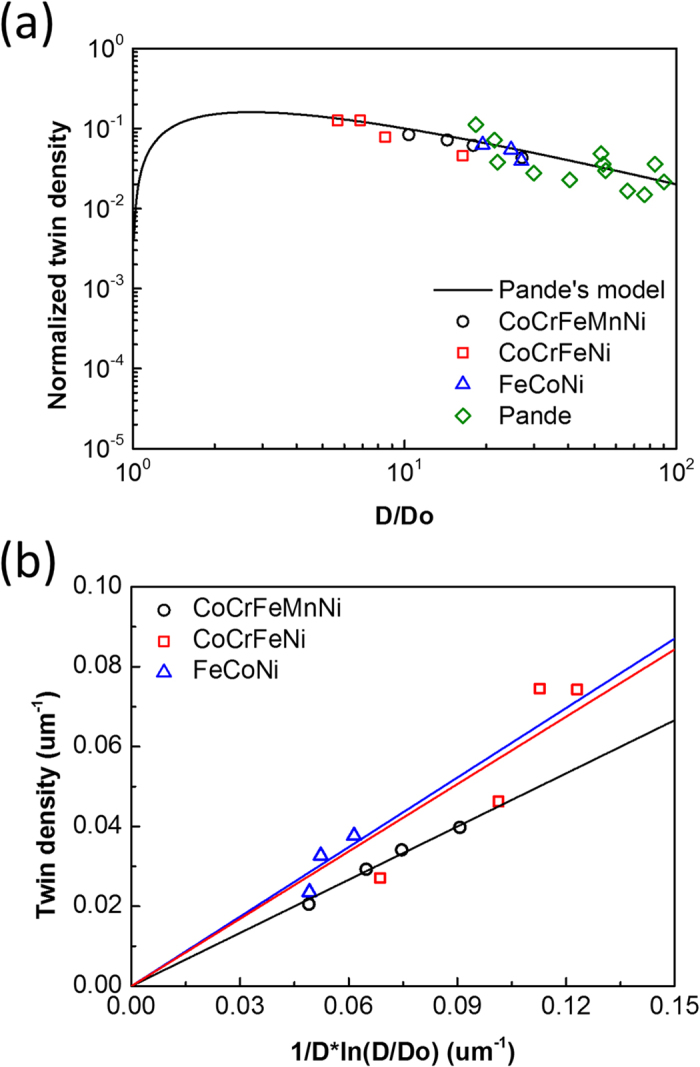
Twin density analysis of CoCrFeMnNi, CoCrFeNi and FeCoNi REC results incluing (**a**) normalized twin density as a function of grain size and (**b**) twin density as a function in Pande’s model.

To further analyze and discuss the twinning mechanisms for three alloys after REC, the annealing twin formation model, established by Pande *et al.*^15^,^17^,^28^,^29^ was applied:


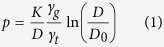


where 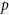 is twin density (number of twin Σ3s intercepted per unit length), 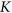 is a dimensionless constant related to strain level, 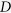 is the average grain size, 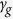 is the average grain boundary energy, 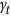 is the twin boundary energy, and 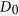 is the critical grain size for annealing twin formation, which can be affected by the step size of EBSD measurement, and is set as 2.487 μm for this study. Figure 5(a) shows the normalized twin density of CoCrFeMnNi, CoCrFeNi, and FeCoNi after REC and Pande’s work^15^, and the data of all alloys coincides with Pande’s model. It should be noted that Pande’s model is derived based on the theory of growth accident, while Σ3 regeneration and special boundary annihilation by HAGB migration are not considered in the derivation. It has yet to be determined whether or not the scattering data can be affected by Σ3-regeneration and special boundary annihilation, but data scattering is clearly shown in Pande’s work. Since there appears to be no correlation between special boundary fraction and twin density after one-step recrystallization in this study. As a result, the effect of Σ3-regeneration and special boundary annihilation is not considered for twin density analysis in present work. Figure 5 (b) shows the twin density of CoCrFeMnNi, CoCrFeNi, and FeCoNi as a function of 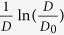. FeCoNi possesses the highest twin density, and CoCrFeMnNi possesses the lowest twin density. It appears that twin density decreases with increasing alloying complexity. This phenomenon can be explained by the average grain boundary energy and twin boundary energy. 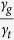 can be expressed as 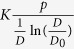, which is equal to 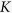 times the slope in Fig. 5(b). FeCoNi possesses a 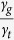 value of 0.580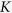, while CoCrFeNi and CoCrFeMnNi have a 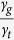 value of 0.562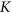 and 0.444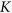, respectively. 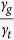 shows a decreasing trend with increasing alloying elements. It is important to point out that data scattering of CoCrFeNi in Fig. 5(b) is well within the data scattering presented by Pande’s growth accident model^15^. The adjusted R-squared of best-fit line in Fig. 5(b) is 0.999 for CoCrFeMnNi, 0.962 for CoCrFeNi, and 0.983 for FeCoNi, and the standard deviation of gradient is 0.00703 for CoCrFeMnNi, 0.0557 for CoCrFeNi, and 0.0438 for FeCoNi, so the change in slope shown in Fig. 5(b) can be taken into account for the following analysis. According to work done by Zaddach *et al.*^22^, Bhattacharjee *et al.*^21^, and Wu *et al.*^24^, it can be concluded that stacking fault energy is decreased with increasing alloying elements due to both elemental effect and the high energy level of the heavily distorted matrix. Generally, the stacking fault energy is about twice the twin boundary energy^30^, hence 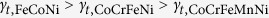, which indicates that HEA needs less energy for twin formation. However, when considering the value of 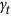 and 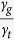, a ranking of average grain boundary energy as 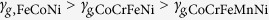 can be deduced, which is an indication of the driving force for grain growth. However, 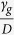 may be further decreased by alloying, e.g. the Cr content in CoCrFeNi is higher, to decrease the driving force for grain growth in CoCrFeNi. It should be noted that the average grain boundary energy of CoCrFeMnNi can be affected by the presence of Mn-Cr oxides, because these particles may hinder grain boundary migration^31^. These results suggest that the elemental effect and lattice distortion can decrease both the driving force for grain growth and twin boundary energy. As a result, twin density of CoCrFeMnNi is lower than the other two after REC.

This study analyzed the effect of GBE on FCC HEA system with alloying complexity ranging from 3 to 5 elements, and the results show that the alloying element and its amount can influence GBCD significantly. To populate higher fractions of special boundaries by one-step recrystallization process, alloying design should aim to induce high grain boundary velocity and high grain boundary energy, while elements like Mn, which form particles in the alloy to impede grain boundary migration, should be avoided. Future work will include the iterative strain annealing process, the results of which will provide further insights on the correlations between grain boundary velocity, strain consumption rate, and evolution of GBCD in complex alloy systems.

## Conclusion

Grain boundary evolutions of equiatomic CoCrFeMnNi, CoCrFeNi, and FeCoNi were studied after one-step recrystallization. Among these three tested alloys, FeCoNi exhibited the highest special boundary fraction and twin density after one-step recrystallization. The underlying mechanisms are summarized below:

1. Boundary interaction mechanism and special boundary fraction depend on the grain boundary velocity, which is mainly affected by the alloying element in this study, and the special boundary population is mainly controlled by HAGB dissociation.
2. Twin density fits well with Pande’s model, because the main boundary interaction mechanism is HAGB dissociation (growth accident), which is the foundation of Pande’s model.
3. Twin density decreases with increasing amounts of the alloying element, because the alloying effect and severe lattice distortion can decrease both the average grain boundary energy and twin boundary energy; the effect of decrease in average grain boundary energy is more pronounced than that of the decrease in twin boundary energy.

## Methods

Samples of equiatomic CoCrFeMnNi, CoCrFeNi, and FeCoNi alloys were melted into rectangular ingots (40.5 × 20.5 × 12.0 mm^3^, 60 g) by arc melting, followed by drop casting within a pure Ar atmosphere. To reduce the impurity level in the CoCrFeMnNi alloy, Mn was cleaned by nitric acid before arc melting in order to minimize oxide contamination^32^. The ingots were arc melted three times to promote chemical homogeneity, and then finally dropped into a rectangular Cu mold. Finally, the rectangular ingots were remelted again to ensure chemical homogeneity. The ingots were encapsulated in quartz tubes with an Ar atmosphere and homogenized for 6 h at 1100 °C, followed by a water quench. After heat treatment, the surfaces of the ingots were ground by #80 grade SiC abrasive papers to remove any surface oxides, and to reduce the thickness of the final ingots to 9.5 mm. Before the GBE process, the ingots of each alloy had an average grain size of 1,000 μm.

The following GBE process was applied to the ingots of each alloy: one-step recrystallization with 50% deformation, followed by recrystallization and water quench. The reduction in thickness was conducted by a DBR-250 rolling mill at ambient temperature. The specimen was then cut by a wire electrical discharge machine, encapsulated in an evacuated quartz tube and followed by annealing and water quench. The annealing temperature and time were varied to control the grain size of each different alloy.

For metallographic analysis, after each GBE process, the specimens were prepared by a technique proposed by Chen *et al.*^33^ for electron backscatter diffraction analysis. Final polishing was performed using colloidal silica with a 0.02 μm particle size. The polished specimens were analyzed with a Hitachi SU-8010 scanning electron microscope equipped with an EBSD detector under an acceleration voltage of 20 kV. Grain size distribution, remained strain analysis by misorientation distribution method, grain boundary character distribution, and twin density were analyzed with EBSD post-processing software HKL CHANNEL 5^34^. A maximum scanning area of 1.92 × 1.44 mm^2^ was used for grain size distribution, as that size was determined to contain enough grains for statistically valid analysis. The analyzed area of special boundary was specified with 22 × 18 *D*^2^, wherein *D* is equal to average grain size. Step size was defined between 1.243 μm and 4.145 μm, depending on the average grain size of the specimens. The rolling direction of the analyzed area is from left to right, while the transverse direction is from bottom to top. A critical misorientation of 10° was used for grain size determination. Σ3 boundaries with θ < 5° were not counted as grain boundaries for grain size determination. These were the default settings of HKL CHANNEL 5 software. Grains detected with only three pixels or lower were removed to reduce noise^35^. The grain aspect ratio was calculated by the ratio of grain length to the grain width.

Remained strain of alloy after annealing was estimated by misorientation distribution, which was analyzed by the recrystallized fraction component of HKL CHANNEL 5 software. If the internal misorientation in a grain exceeded the minimum angle to define a subgrain, which was defined as 2°, the grain was classified as a deformed grain. If a grain was composed of subgrains whose internal misorientation were under 2° but the misorientation from subgrain to subgrain was above 2°, the grain was classified as a substructured grain. The remaining grains were classified as free of strain (undeformed grains). CSL boundaries were defined by Brandon’s criterion: 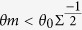, where 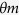 is the maximum permissible deviation from coincidence and 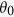 ~ 15° 36. Twin Σ3 boundaries were distinguished from non-twin Σ3 boundaries by 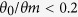, where 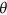 is the permissible deviation from coincidence^7^. Twin density, defined as the number of intercepts per unit length, was calculated by the following equation: 
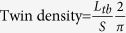
, where 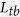 is the twin Σ3 boundary length and 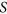 is the corresponding area^37^.

## Additional Information

**How to cite this article**: Chen, B.-R. *et al.* Effect of one-step recrystallization on the grain boundary evolution of CoCrFeMnNi high entropy alloy and its subsystems. *Sci. Rep.* **6**, 22306; doi: 10.1038/srep22306 (2016).

## Figures and Tables

**Table 1:** Composition of Mn-Cr oxides.

	**Co**	**Cr**	**Fe**	**Mn**	**Ni**	**O**
Atomic%	0.42	29.44	0.46	15.43	0.58	53.67

**Table 2:** Annealing condition, grain size analysis, remained strain analysis, and special boundary fraction of one step recrystallization (REC).

REC results												
Composition	Annealing condition	Grain size		Remained strain analysis			Special boundary fraction					
		*D* (μm)	Stdev. (μm)	Und. (%)	Sub. (%)	Def. (%)	Twin Σ3 (%)	Nontwin Σ3 (%)	Σ9 (%)	Σ27 (%)	Other CSL (%)	LAGB (%)
CoCrFeMnNi	900 °C/1 h	25.8	19.8	95.13	4.82	0.05	47.1	2.39	2.09	0.32	3.06	4.50
	950 °C/1 h	35.7	22.8	94.19	5.62	0.19	42.1	3.87	1.16	0.12	3.02	5.86
	1000 °C/1 h	44.5	29.4	95.08	4.82	0.10	45.1	4.21	1.20	0.39	2.84	6.34
	1050 °C/1 h	67.3	44.5	96.83	3.12	0.05	46.9	3.56	0.65	0.13	2.73	5.96
CoCrFeNi	800 °C/2 h	14.1	10.1	87.15	12.60	0.25	44.3	3.02	3.01	0.73	2.12	4.04
	800 °C/6 h	17.1	13.3	90.21	9.68	0.11	47.4	2.23	2.83	0.87	3.29	5.21
	1100 °C/1.5 h	21.1	13.7	88.26	11.19	0.55	36.3	4.47	1.41	0.64	3.45	7.37
	1100 °C/2 h	40.7	26.3	95.00	4.96	0.04	39.4	3.87	1.51	0.33	3.03	6.31
FeCoNi	800 °C/20 min	48.2	45.0	86.99	12.97	0.04	54.9	2.03	4.52	1.74	2.25	2.36
	800 °C/30 min	61.3	53.0	90.00	9.99	0.01	57.7	1.95	3.42	1.18	2.08	2.18
	700 °C/1 h	67.0	77.1	85.02	14.94	0.04	61.5	1.95	5.55	1.74	2.13	2.12

Abbreviations are average grain size (*D*), standard deviation (Stdev.), undeformed grains (Und.), substructured grains (Sub.), and deformed grains (Def.), low angle grain boundary (LAGB).
